# Epidemic, or Army Itch

**Published:** 1866-03

**Authors:** L. C. Butler

**Affiliations:** Essex, Vt.


### ON EPIDEMIC, OR ARMY ITCH.

By L. C. BUTLER, M.D., Essex, Vt.

Perhaps I cannot better respond to the request of your correspondent in the *Reporter* for Jan. 6th, than by sending a copy of the Report made to the Vermont Medical Society, with such emendations and alterations as may be necessary to adapt it to your columns. I was not aware, till since the publication of the proceedings of our State Society in the *Reporter*, that the disease in question was so wide-spread in its ravages. It seems to be limited in its circumference only by the extent of territory from which recruits were sent to and returned from the Union army. It is not indigenous to northern soil, nor is it the legitimate offspring of any disease known among us. Nor can its history be traced among the archives of medicine. The "oldest" physician does not recognize it as a "familiar" face, and so has little advantage over the younger practitioner in its diagnosis or treatment. It is no respecter of persons. It pays no deference to age, sex, or condition. Pulpit, forum, and bar, learned and unlearned, rich and poor, are all alike the objects of its peculiar friendship. It rides in a coach as cosily as it nestles among the rags of poverty. It sits as jauntily upon the luxurious sofa in silks and satins, as upon the hard floor. It sleeps as quietly (?) upon nature's bed and under nature's broad covering, as upon the downy couch and behind damask curtains. It seems to recognize no known law of incubation, progress, or termination. That it is contagious, highly so, there is no doubt. It is somehow communicated from one to another. Sometimes from actual contact, and sometimes without; sometimes as rubeola and scarlatina, and sometimes more as an epidemic. Its progress is better defined by the intolerable itching and scratching it occasions.

So far as its appearance in these parts is concerned, it can be traced almost invariably to southern camping grounds, whence it has been transplanted by the tramp of armies, and the return of our brave boys from the "sacred soil" to their various homes. It is not a native "to our manor born." It is an importation, a peculiar child of southern generation.

In regard to its nomenclature, possibly the persevering student of Good may find in that stupendous compendium of nosology, and nosological infinitudes, some huge Latin name applied to it; but the practitioner who has seen it and treated it will be far better able to say what it is *not*, than what it *is*. It defies both nomenclature and classification. Taking Néligan as our standard, it does not belong to the *Exanthemata*. It exhibits no inflammatory tendency. Nor to the *Pustulæ;* it has no pustules. Nor to the *Squamæ*, for it produces no secretion of any laminated whitish scales on the cutaneous surface. Nor yet to the *Hypertrophæ*, or *Hemorrhagiæ*, or *Maculæ;* nor does it belong to the *Vesiculæ*. It is not *Scabies*. In this disease there is said to be a delicious satisfaction in hunting out and exposing to the full blaze of daylight, the ugly *crab*bed parasite, pathognomonic of it, that covers its burrowing place in the skin under a flood of water. Progress has been made in relieving the world of a pest, and momentary relief is obtained. But no such satisfaction is vouchsafed to the unfortunate victim of "army itch." It is scratch, scratch; the more, the greater necessity; wriggle and twist "from night till hoary morn;" for the itching is quite tolerable during the daytime, but most intolerable during the night.

In many of its characteristics it corresponds most nearly with the *Papulæ*, and with the genus *Prurigo;* and yet that name gives only an inkling of some of its peculiar symptoms, while it affords no therapeutic indication.

Unlike *prurigo*, this disease is highly contagious. It has no vesicle, nor has it any pustule, yet sometimes the eruption, from the incessant irritation it occasions, forms a discharging surface, upon which scabs are formed. It seldom (in my opinion never) in its inception, is found in the groins, axilla, armpits, or between the fingers. It is always found upon the arm, forearm, chest, abdomen, or lower extremities, and, in some rare cases, upon the scalp. Sometimes it is a fine eruption, about the size of a millet seed, hardly discoloring the skin, or raised above it; then, again, it resembles rubeola, and gives a marked sensation of roughness to the cutaneous surface. The attending pruritus is sharp and stinging, causing almost incessant scratching, by which the papulæ are torn, and a minute blackish crust formed on their apices, giving the eruption a peculiar characteristic appearance. Underneath this crust is a minute red point, which fades away as a new crop makes its appearance. In long-continued cases, complicated with other skin diseases, or accompanied with severe constitutional symptoms, suppurating sores may be formed upon different portions of the body, which will test the patience of both physician and patient in their cure.

Whilst, therefore, it so nearly corresponds with the description given in the books on *prurigo*, still the remedies ordinarily prescribed for that disease will produce little, if any, curative effect in this. It is, in fact, *sui generis*, and no better name can be found for it than that which has been given to it in common parlance, from its recognized origin, "*Army Itch.*" And yet, this only represents its outward manifestation, the cutaneous affection. Back of this, and perpetuating the disease, is the *materies morbi*, which the force of every pulsation sends through the whole system. To cure the one, the other must be eradicated. Constitutional as well as local remedies must be employed. The latter are useless without the former.

In accordance with these views, I begin and end the treatment with constitutional as well as local remedies, as follows:—

℞. Syr. Sarsap. Comp., ---------- f.℥iv.

Sodæ Arsenias, ---------------- gr. iv.—viij.

M. Dose, teaspoonful, morning and evening.

To this mixture, if there be considerable derangement of the stomach or biliary organs, I add, *pro re nata*, Thayer's fluid extract of iris versicolor, leptandrin, pipsissewa, or podophyllin as seems best adapted to the case. The external application is composed as follows:—

℞. Pix Burgundica, ---------------------- ℥iv.

Hydrarg. Oxyd. Rub., ---------------

Plumbi. Oxyd. Rub.,-----------------

Terebinth Veneta,-------------------āā ℥j.

Butyri Recentis,--------------------- ℥xij.

M. (Adeps will not answer.)

The first three ingredients should be finely pulverized before being mixed with the last two, and then constantly stirred over a slow heat until all are intimately blended. Then place the dish upon ice or cold water, and stir again until the whole becomes the consistence of an ointment. A small portion of this ointment should be rubbed in thoroughly upon the eruption, morning and evening.

Great care should be observed in regard to cleanliness. The under clothing should be changed twice or thrice a-week, and an occasional ablution of the whole body with Castile soap and water, would not only tend to allay the cutaneous irritation, but expedite the cure. The diet should be carefully regulated. No pork grease in any form should be allowed. Pastry, highly seasoned food of any kind, stimulating condiments, should be avoided as directly calculated to perpetuate the disease. The patient should not be starved, but the diet restricted to those articles which are plain, simple, and nourishing, and which will not tax too heavily the digestive organs.

The course of treatment I have thus indicated, is the result of some investigation and observation. It has been adopted after a thorough trial and failure of many prescriptions. In my hands, it has been uniformly successful; I can call to mind no case which, when the directions have been carefully followed, has not been cured by it. Time, patience, and perseverance are necessary to accomplish the result, but it is sure. Recent cases are more speedily cured than those of longer duration. I write with the more confidence, because I have tested the treatment in hundreds of cases, and have yet to see the instance of failure. I prepare the remedies myself. I do not deal out the ingredients to the patients, and leave them to prepare the ointment with such grease as they may happen to have. The prescriptions are compounded carefully by weight and measure, and just the heat necessary for their intimate mixture. And, having taken all these precautions, which I regard as important, I watch the progress of the cure, and am not disappointed in finding the intolerable itching allayed, so that the patient will enjoy a quiet night, in many instances, from the first application, and the eruption fading away and disappearing. In other localities, or in the hands of other practitioners, they may not prove as uniformly successful, but where other remedies have failed, these are spread out before the profession as being worthy, at least, of a thorough trial.—*Med. & Surg. Reporter.*

